# Effects of long-term integrated agri-aquaculture on the soil fungal community structure and function in vegetable fields

**DOI:** 10.1038/s41598-021-90109-6

**Published:** 2021-05-24

**Authors:** Xianqing Zheng, Ke Song, Shuangxi Li, Hanlin Zhang, Naling Bai, Juanqin Zhang, Haiyun Zhang, Shumei Cai, Weiguang Lv, Linkui Cao

**Affiliations:** 1 School of Agriculture and Biology, Shanghai Jiao Tong University, Shanghai, 200240 China; 2 Institute of Eco-Environment and Plant Protection, Shanghai Academy of Agricultural Sciences, Shanghai, 201403 China; 3 National Agricultural Experimental Station for Agricultural Environment, Fengxian, Shanghai, 201403 China; 4 Shanghai Key Laboratory of Protected Horticultural Technology, Shanghai, 201403 China

**Keywords:** Agroecology, Microbial ecology, Fungal biology

## Abstract

The diversity and community structure of soil fungi play an important role in crop production and ecosystem balance, especially in paddy-upland vegetable field systems. High-throughput sequencing was used to study changes in the soil fungal community structure and function in paddy-upland vegetable field systems. The results showed that compared with traditional planting, the diversity and community structure of soil fungi were changed by the combination of flooding and drought, the Shannon index increased by 11.07%, and the proportion of the dominant species, *Mortierella*, decreased by 22.74%. Soil available nitrogen, total phosphorus, available phosphorus, total nitrogen and organic matter played a leading role in the initial stage of the experiment, while the dominant factor changed to total potassium 3 years later and then to soil pH and water content 6 years later. FUNGuild analysis showed that the proportion of three independent trophic modes of soil fungi were increased by the combined flooded-drought model, and there were multiple interaction factors, For example, nutrient supply, pH and planting pattern. This study showed that soil fertility, crop yield and economic benefits were better than the traditional model after three years of planting and breeding. The longer the time, the better the effect.

## Introduction

To alleviate the pressure of the growing world population on food demand, measures increasing the multiple cropping index of cultivated land and the inputs of chemical pesticides and chemical fertilizers have been adopted worldwide. Under this scenario, in the long run, the quality of the farmland soil environment will deteriorate^1,2^, the diversity of the soil microbial community will decrease^3,4^, and the quality of agricultural products will also decrease. Previous results show that the combination of planting and rearing animals can increase soil nutrient content, reduce farmland pollution load, improve soil quality and the environment, and improve soil microbial Community, all of which are beneficial for the development of green agriculture in the future^5–8^. The results showed the intercropping patterns under forest cultivation and farmland intercropping in dryland systems, such as forage crop-cow farming models, symbiotic paddy field systems, such as rice-fish, rice-duck and rice-frog models, and mixed rice-upland farming systems, such as grain-vegetable-pig, rice-mushroom-goose and vegetable-eel-earthworm combinations^9–11^. The soil microbial community, especially the soil fungal community, plays an important role in the processes of these models. When human beings use these production models to transform and regulate the farmland system to obtain high-quality food resources, it will also affect the soil ecological environment, including soil fungal communities.

Soil fungi are an important component of the soil microbial community and are widely distributed within the soil system. Soil fungal community composition and function are of great significance for maintaining the stability of agroecosystems^12^. Together with other soil organisms, soil fungi are involved in soil formation and development, organic matter cycling, energy flow, and fertility changes in agroecosystems^13,14^. These processes include organic matter decomposition and synthesis in the soil, nutrient transformation and cycling, biological control, pollution remediation^15–17^, etc. It has been shown that compared with bacteria, the enzymes secreted by soil fungi play a more important role in the decomposition and transformation of complex compounds such as cellulose, lignin, and pectin^18^ and directly influence the amount and composition of soil organic matter^19^. Many soil fungi, including *Trichoderma* spp., can be used for the biological control of plant diseases and crop pests^20,21^. Under natural conditions, soil fungi have a large biomass, high survival and reproductive capacity, and stable genetic traits, and there are strains that are efficient for treating large areas that have been polluted by organophosphate pesticides^22^. As the state of the soil mycoflora can greatly influence the productivity and ecological balance of farmlands, these organisms are a key indicator of soil quality^23^. Therefore, many researchers have studied how the soil environment, nutrients, and biological factors can be regulated to influence soil fungal community structure and function to generate positive feedbacks in agroecosystems and to facilitate the sustainable development of green agriculture^24–26^. Adding fertilizers, organic materials, and modifiers to farmland soil to alter soil nutrient distribution and pH is a common measure that is used to regulate soil mycoflora^27^. Mandić et al. (2004) found that the type, amount, and timing of fertilizer applications significantly affected soil fungal abundance. Organic and mineral fertilizers can increase soil fungal abundance in maize fields, alter the community composition, enhance the activity of maize rhizosphere arbuscular mycorrhizal fungi (AMF), and facilitate interactions between AMF and other species^28^. Applying horse manure to soil can introduce additional fungi^29^. Adding wood ash to forest soil can strongly affect fungal respiration and growth rates^30^. Soil moisture content is also a key factor controlling soil fungal abundance, and a relative water content of 60% is a critical value. When the relative moisture content is below 60%, soil fungal abundance increases with water availability; in contrast, when the relative moisture content is above 60%, soil fungal abundance decreases with water availability. Humans also regulate soil fungal communities through biological means, e.g., allowing grazing (by cattle) on human-managed grasslands can increase the number of fungal colonies in grassland soil^31^. Studies have also examined how planting systems regulate soil fungal community richness, structure, and function. Chen et al. (2015) chose limestone yellow soil in northern Guizhou Province to conduct a 10-year continuous cropping and intercropping experiment on flue-cured tobacco, wheat, *Brassica rapa*, and maize^32^. Liao et al. (2019) found that rice-duck integrated planting and breeding increased the abundance of *Ascomycota* by 2.21 times at the phylum level and the abundance of *Melanconiella* by 25.62 times at the genus level and reduced the relative abundance of *Chaetomium* and *Mycothermus* by 10.12 and 44.20 times, respectively^33^. In addition, recent studies suggest that soil fungi may stimulate inactive bacteria and may determine the future of soil carbon^34,35^.

To sum up, soil fungi play an important role in crop production and farmland ecosystem maintenance. There have been many studies on the abundance, structure, diversity, regulation and utilization of soil fungi in different types of farmland ecosystems. However, soil fungal community structure and function under combined cultivation systems and the influence of various factors in the system on the spatial and temporal scales have not been reported. In order to develop and use soil fungi to serve the flooded and dry/upland co-farming system to improve farmland production and economic benefits, in this study, a model of rice-eel-vegetable-earthworm production coupled with paddy upland farming in vegetable fields was established, and the temporal and spatial variation of soil fungal community structure and diversity and its influencing factors were discussed.

## Results

### Effects of the two planting systems on soil fungal diversity

**Figure 1:**
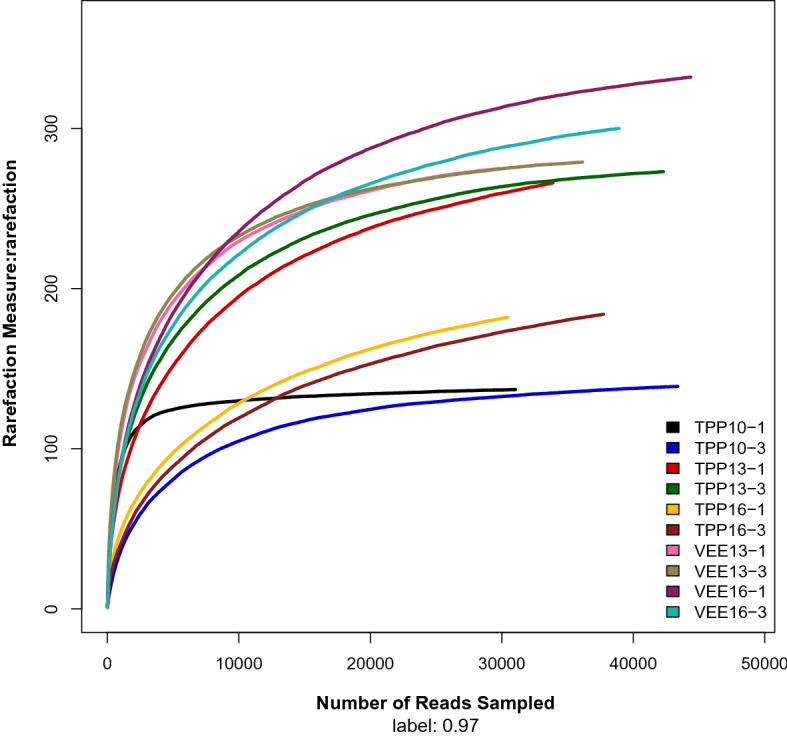
α-Diversity comparison. Rarefaction curves for OTUs were calculated using Mothur (v1.27.0) with reads normalized to more than 30,000 for each sample using a distance of 0.03 OTU.

In this study, 561,254 sequences were generated from 15 samples obtained from 5 treatments. Base sequences with a length of 201–300 bp accounted for 97.82% of all sequences (Table S1a,b). Rarefaction curves at a similarity level of 97% indicated that the number of sequences extracted from most samples tended to plateau above 10,000. The number of sequences extracted in the test exceeded 30,000, suggesting that the sequencing data were close to saturation, sequencing depth was reasonable, and the results reflected true sample conditions (Fig. 1). The coverage of all samples was above 99.84%. The range of reads in each sample was between 34,390 and 43,510. The range of Operational Taxonomic Units (OTUs) in each sample was between 145 and 318 (Table 1).

**Table 1:** Comparison of α-diversity indices in TPP and VEE soil samples.

Sample ID	TPP10	TPP13	TPP16	VEE13	VEE16	Rate
Reads	43,510 ± 180.31a	34,390 ± 714.18c	39,150 ± 2004.65abc	37,060 ± 1309.56bc	41,640 ± 3867.87ab	–
OTUs	145 ± 11.59d	273 ± 7.00b	198 ± 26.58c	274 ± 5.03b	318 ± 16.26a	56.94
Chao	150 ± 11.37d	291 ± 10.79b	242 ± 27.84c	290 ± 0.00b	338 ± 11.14a	33.81
Coverage	0.9998 ± 0.0001a	0.9990 ± 0.0005bc	0.9986 ± 0.0001c	0.9992 ± 0.0002b	0.9990 ± 0.0001bc	32.50
Shannon	2.04 ± 0.1626a	2.92 ± 0.0141a	2.14 ± 0.0354a	3.23 ± 0.1202a	2.53 ± 0.0707a	21.86
Simpson	0.39 ± 0.4842a	0.13 ± 0.0057a	0.23 ± 0.0113a	0.10 ± 0.0078a	0.21 ± 0.0095a	− 11.67

“Rate” refers to the growth rate of VEE16 over TPP16.

Means followed by the same letter a, b or c indicates there is no significant difference among the treatments at *p* < 0.05 (n = 3), Number after “+/−” represents the standard deviation. TPP means a traditional planting pattern while VEE means a vegetable–eel–earthworm integrated planting and breeding platform.

The analysis of alpha diversity showed that with increasing planting time, soil fungal OTUs, the Chao index, and the ACE index in TPP-treated plots increased and then decreased with time. In the VEE-IPBP-treated plots, these 3 indexes increased with time and were 56.94%, 33.81%, and 32.50% higher than those in the TPP-treated plots, respectively, after 6 years of implementation (*p* < 0.05). Under both planting systems, the Shannon index values of soil fungi increased and then decreased with time; however, within the same period of time, these values were 21.86% and 11.07% higher in the VEE-IPBP-treated plots than in the TPP-treated plots after 3 and 6 years, respectively. This indicated that VEE-IPBP led to greater increases in soil fungal richness and diversity than TPP (Table 1).

### Comparison of soil fungal community structure between the two planting systems

**Figure 2:**
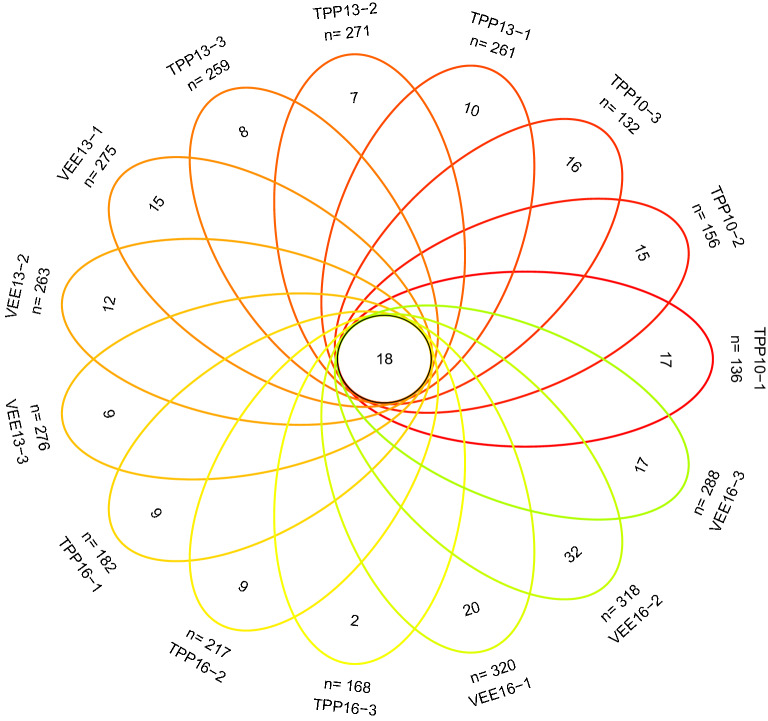
Venn diagram of the number of common and unique operational taxonomic units (OTUs). The number in the circle of petal graph represents the number of similar OTUs among samples, and the number after n represents the number of OTUs contained in a sample alone.

Venn diagrams showed that the number of fungal OTUs that were common across samples from the TPP- and VEE-IPBP-treated plots across all planting time scales was 18 (Fig. 2). There were relatively few OTUs unique to the TPP-treated plots across all planting time scales; there were 25 and 20 in TPP13 and TPP16, respectively. There were more OTUs unique to the VEE-IPBP-treated plots across all planting time scales, with 36 and 69 for VEE13 and VEE16, respectively, and they exhibited an increasing trend. This was consistent with the trend in the total number of OTUs, indicating that VEE-IPBP altered the soil fungal community structure.

**Figure 3:**
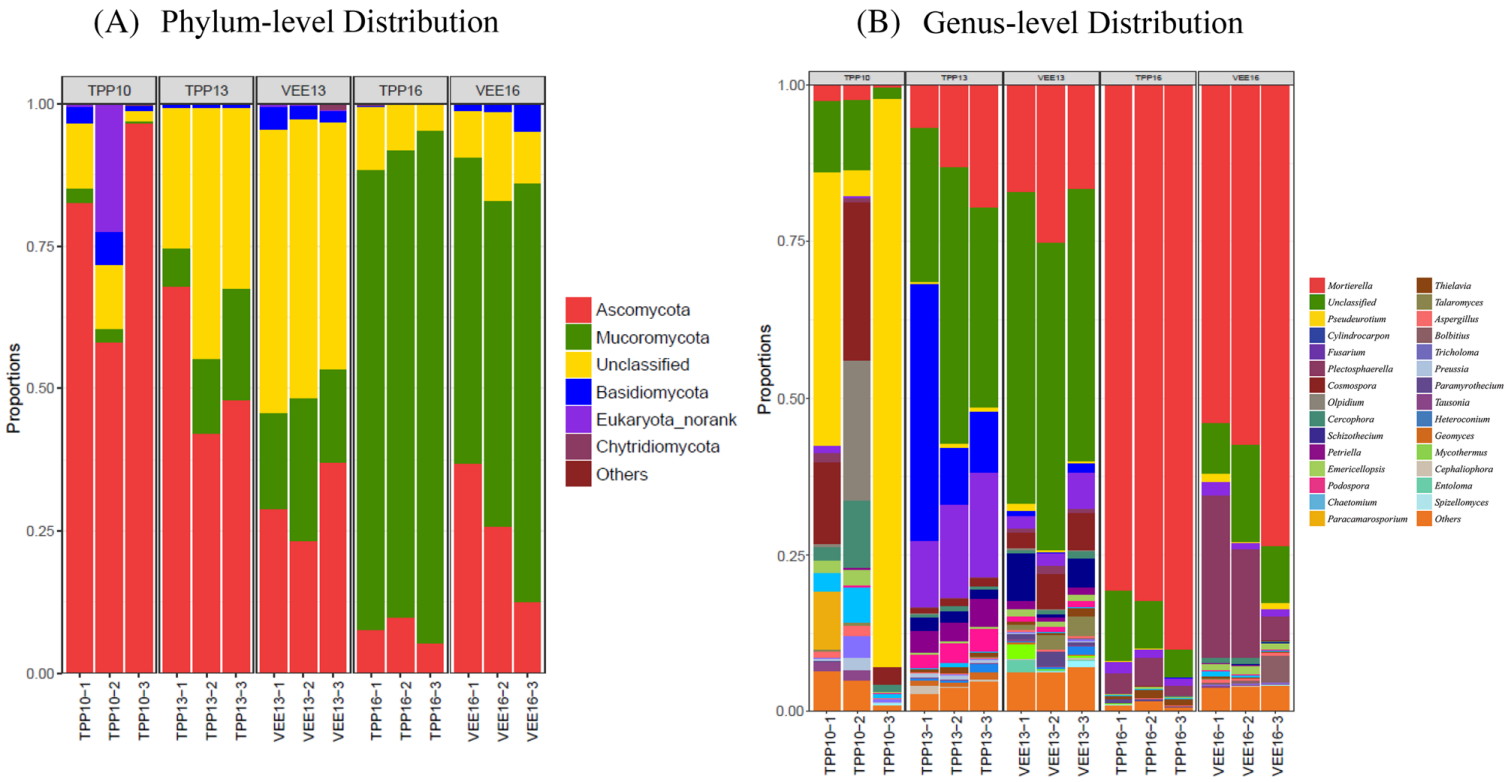
Analysis of the soil fungal community structure and composition in plots subjected to different planting systems. (**A**) Community structure and composition at the phylum level; (**B**) community structure and composition at the genus level. Taxa with an abundance of less than 1% were combined into the category ‘other’.

The analyses show that soil fungal diversity significantly increased with the duration of the experiment. There were significant differences in soil fungal abundance between the TPP- and VEE-IPBP-treated plots. At the phylum level (Fig. 3a), there were 5 fungal phyla with an abundance greater than 1% in the two planting systems: Ascomycota, Mucoromycota, Basidiomycota and Chytridiomycota. At the beginning of the experiment, in TPP10, Ascomycota were the dominant taxon, accounting for 79.21% of the fungal abundance. As the experiment progressed, Ascomycota declined rapidly, accounting for 52.70% of the abundance in TPP13 after 3 years of implementation and 7.62% in TPP16 after 6 years of implementation. Ascomycota also showed a decreasing trend in the VEE-TPB-treated plots, but it was maintained at a certain level in the later period. Ascomycota accounted for 29.70% of the abundance in VEE13 after 3 years of implementation and 25.04% in VEE16 after 6 years of implementation. Mucoromycota became the dominant taxon, accounting for 84.32% and 61.58% of the abundance in TPP16 and VEE16, respectively.

At the genus level (Fig. 3b), it was also shown that different plant systems altered the dominance of soil fungal taxa. At the beginning of the experiment, the genera *Pseudeurotium*, *Cosmospora*, *Olpidium*, and *Cercophora* were the dominant taxa, together accounting for 80.36% of the abundance. As the experiment progressed, the dominant taxa in the TPP-treated plots 3 years after implementation were *Cylindrocarpon*, *Fusarium*, *Mortierella*, and *Petriella*, accounting for 84.34% of the abundance. The dominant taxa in the VEE-TPBP-treated plots were *Mortierella*, *Cosmospora*, *Schizothecium*, and *Fusarium*, accounting for 79.12% of the abundance. The dominant taxa in the TPP-treated plots 6 years after implementation were *Mortierella*, *Plectosphaerella*, *Fusarium*, and *Thielavia*, accounting for 97.60% of the abundance. Among these taxa, *Mortierella* was the most dominant, accounting for 84.32% of the abundance. The dominant taxa in the VEE-TPBP-treated plots were *Mortierella*, *Plectosphaerella*, *Bolbitius*, and *Fusarium*, accounting for 91.23% of the abundance. Although *Mortierella* was again the most dominant taxon, it only accounted for 61.58% of the abundance.

### Differences in soil fungal communities between the two planting systems

**Figure 4:**
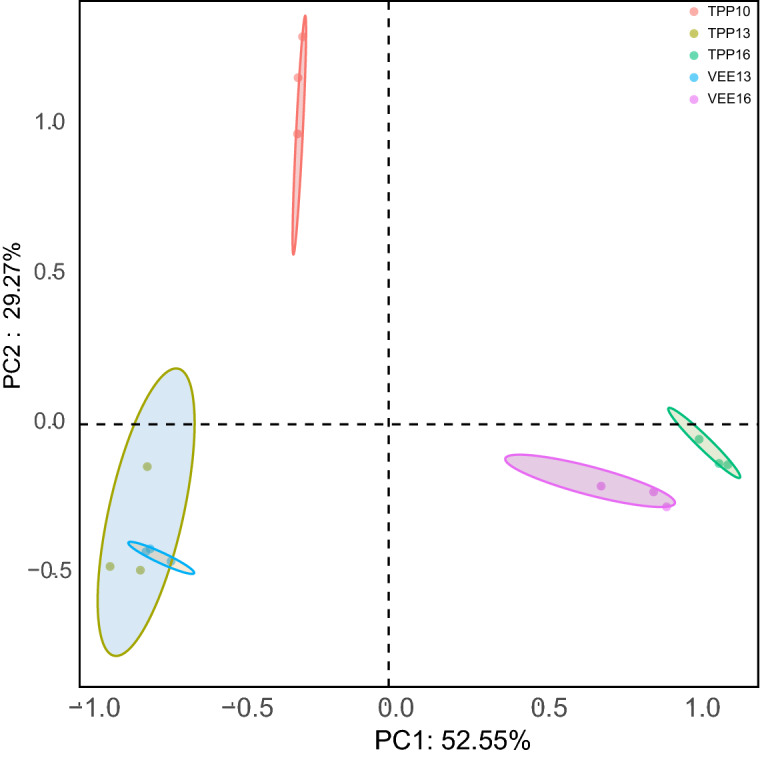
PCoA of the influence of two planting patterns on the diversity of soil fungi.

The results of PCoA analysis showed that the communities in the samples of the two planting systems had relatively discrete distributions across different time scales with relatively large distances between the samples (Fig. 4). As the experiment progressed, the soil fungal community diversity in the TPP- and VEE-IPBP-treated plots underwent changes. The communities in TPP13 and VEE13 after 3 years of planting showed a tendency to diverge, and after 6 years of planting, the communities in TPP16 and VEE16 had completely diverged. This indicated that differences in the planting system could influence the separation distance of the soil fungal community structure.

**Figure 5:**
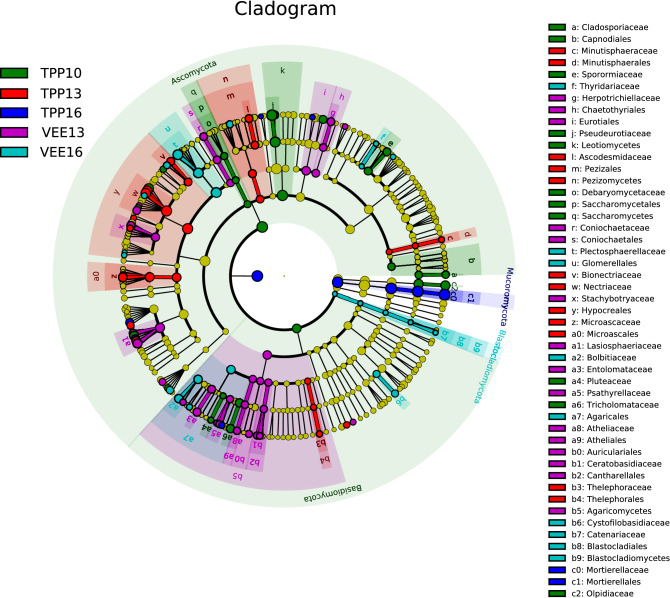
Analysis of the differences in the composition of the fungal community between the two planting patterns (LDA scores greater than 2).

The results of the LEfSe analysis (Fig. 5) showed that there were 117 taxa with an LDA score greater than 2 in the 15 samples from 5 treatments that were collected at 3 time points from the 2 planting systems. The distribution of taxa across 5 taxonomic levels was as follows: 4 at the phylum level, 5 at the class level, 17 at the ordinal level, 27 at the family level, and 63 at the genus level. At the phylum level, 2 taxa of soil fungi in TPP10 exhibited relatively high relative abundance: Ascomycota and Basidiomycota. As time progressed, these highly abundant taxa underwent changes with differences between the two planting systems; 6 years after implementation, the dominant taxa were Mucoromycota in TPP16 and Blastocladiomycota in VEE16. There were significant differences (*p* < 0.05) between the two planting systems at all taxonomic levels.

At the genus level, the taxa that differed between samples underwent changes in abundance over time and differed between planting systems. There were 12 such taxa in TPP10 (belonging to the genera *Pseudeurotium*, *Cosmospora*, *Olpidium*, *Cercophora*, and *Tricholoma*), 14 in TPP13 (*Cylindrocarpon*, *Fusarium*, *Petriella*, *Podospora*, and *Geomyces*), 6 in TPP16 (*Mortierella*, *Thielavia*, *Cladorrhinum*, *Psathyrella*, and *Thermomyces*), 18 in VEE13 (*Schizothecium*, *Paramyrothecium*, *Entoloma*, *Heteroconium*, and *Volutella*), and 13 in VEE16 (*Plectosphaerella*, *Bolbitius*, *Tetracladium*, *Cystofilobasidium*, and *Ascorhizoctonia*).

### Relationships between environmental factors and between and among fungal communities in the two planting systems

**Figure 6:**
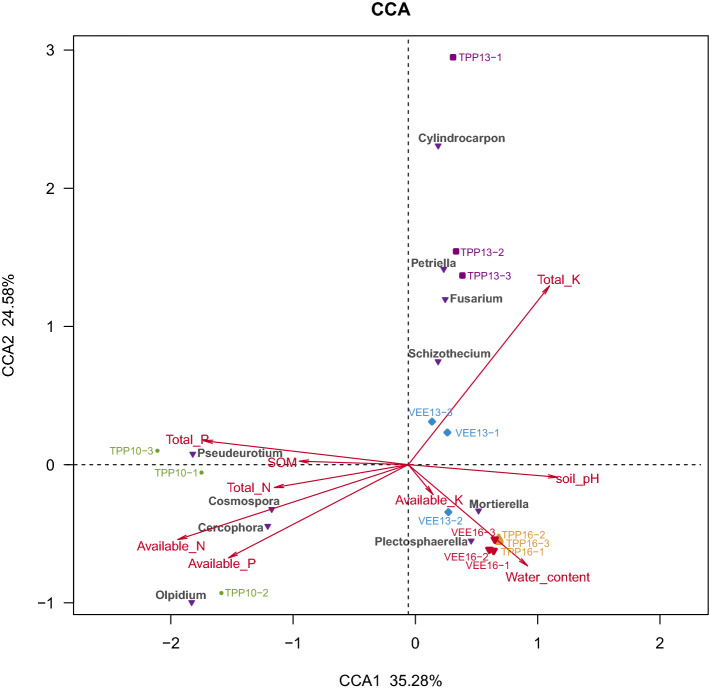
Correlation between soil fungal species and soil physical and chemical properties under different planting patterns and time scales. The abbreviation “SOM” in the figure means “soil organic matter”.

The results of the CCA (Fig. 6) showed that planting systems and time scales explained 59.86% of the relationships between environmental factors and fungal communities. In TPP10, soil fungal taxa were mainly influenced by soil nutrients and had the highest correlation with soil available nitrogen followed by soil total phosphorus, available phosphorus, and total nitrogen and had the lowest correlation with organic matter. Soil nutrients mainly affected the genera *Pseudeurotium*, *Cosmospora*, *Cercophora*, and *Olpidium*. After 3 years of implementation, soil total potassium became the key factor and mainly influenced the taxa *Pseudeurotium*, *Fusarium*, *Schizothecium*, and *Cylindrocarpon*. After 6 years of implementation, the soil pH and moisture content became the key factors and mainly influenced *Mortierella* and *Plectosphaerella*.

**Figure 7:**
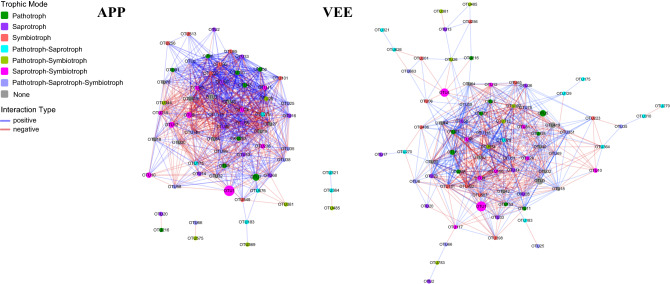
The relationship network of soil fungal trophic types and communities under different planting patterns.

To determine the functions of soil fungi associated with the changes in community structure, The FUNGuild analysis was used to perform functional predictions of soil fungi in vegetable fields under the two planting systems. Based on database annotations, the main trophic modes of soil fungi was established, including pathotrophic (P), saprotrophic (Sa), and symbiotrophic (Sy), as the 3 independent trophic modes and multitrophic modes with two or more trophic modes. The 3 independent trophic modes were further divided into 12 subgroups. A total of 78.0% of all trophic mode predictions were explained, 18.7%, 24.3%, and 0.4% of which were accounted for by the 3 independent trophic modes (P, Sa, and Sy, respectively). The 4 multitrophic modes, pathotrophic-saprotrophic (P–Sa), pathotrophic–symbiotrophic (P–Sy), saprotrophic–symbiotrophic (S–S), and fully mixed (P–S–S) trophic modes, accounted for 0.2%, 0.5%, 47.2%, and 8.7% of the predictions, respectively. Complex direct and indirect positive and negative interactions existed between fungal taxa within and between trophic modes, forming a complex network of taxonomic relationships (Fig. 7).

Compared with TPP, the long-term VEE-IPBP treatment increased the proportions of the 3 independent trophic modes and 2 multitrophic modes (P–Sa and P–Sy) of soil fungi and reduced the proportions of S–S and fully mixed trophic modes. The subgroups of the trophic modes of soil fungi underwent changes accordingly. For example, among the pathotrophic fungi, plant pathogens accounted for 94.4% of fungi in the TPP-treated plots, and *Cylindrocarpon* and *Plectosphaerella* had relatively high abundances. Fungal parasites accounted for 5.0% of fungi, with *Cosmospora* being the main taxon. In contrast, in the VEE-treated plots, the proportions of plant pathogens were reduced to 77.6%, and *Plectosphaerella* and *Cylindrocarpon* had relatively high abundances. The proportion of fungal parasites increased to 19.3%, and *Cosmospora* remained the main taxon. Coprophilous fungi accounted for 42.0%, with *Thielavia* and *Schizothecium* being the dominant taxa. Soil saprophytic fungi accounted for 5.8%, with *Geomyces* being the dominant taxon. In contrast, in the VEE-treated plots, the abundance of *Geomyces* was reduced to no more than 0.7%. The dominant taxa of coprophilous fungi (accounting for 41.0%) became *Schizothecium*, *Bolbitius*, and *Cercophora*. For symbiotrophic fungi, only endomycorrhizae and ectomycorrhizae were detected in the two planting systems. The dominant taxa were *Lecythophora* and *Tomentellopsis* in the TPP-treated plots and *Leptodontidium* and *Hebeloma* in the VEE-IPBP-treated plots. For the S–S mode, which had a relatively high abundance overall, lower abundances of saprotrophic and ectomycorrhizal *Peziza* endophytic *Acrostalagmus* were detected in the VEE-IPBP-treated plots than in the TPP-treated plots.

Due to the multiple influences of environmental factors, nutrient sources, and biological factors, soil fungal taxa form a complex network of interactions. However, the relationship networks of the same taxa differed among planting systems. For example, in the TPP-treated plots, there were 34, 28, 57, and 7 fungal taxa that were positively correlated (+) with the 4 highly abundant and representative taxa *Cylindrocarpon*, *Plectosphaerella*, *Cosmospora*, and *Schizothecium*, respectively. There were 32, 51, 16, and 5 taxa that were negatively correlated with these representative taxa, respectively. In the VEE-IPBP-treated plots, 41, 48, 40, and 24 taxa were positively correlated with *Cylindrocarpon*, *Plectosphaerella*, *Cosmospora*, and *Schizothecium*, and 45, 37, 41, and 11 taxa were negatively correlated with these representative taxa, respectively. The fungal taxa that were the most positively (+) and most negatively (−) correlated with these representative taxa differed between the planting systems. In the TPP-treated plots, the fungal genera that were most strongly correlated with the aforementioned 4 representative fungal taxa were *Ascodesmidaceae* (+) and *Acrophialophora* (−), *Mortierellaceae* (+) and *Psathyrella* (−) *Schizothecium*, and *Mycosphaerellaceae* (−). In the VEE-IPBP-treated plots, the fungal genera that were most strongly correlated with the aforementioned 4 representative fungal taxa were *Curvularia* (+) and *Lectera* (−), *Lectera* (+) and *Curvularia* (−), *Podospora* (+) and *Ascorhizoctonia* (−), *Eurotiales* (+) and *Acremonium* (−) (see Figs. 6, 7).

## Discussion

In this study, an open, fixed-location experiment was established in small plots within fields. Through multiyear, regular sampling, this research aimed to determine differences in soil fungal community structure and function under natural conditions between the VEE-IPBP- and TPP-treated plots and changes in their patterns over time. The results showed that compared with TPP, VEE-IPBP enhanced soil fungal abundance and diversity indices. This could be due to the addition of earthworms to the soil. Earthworms accelerate the transformation of soil organic matter to available nutrients and stimulate microbial activity. Our results were consistent with the conclusion by Wang et al. (2015) regarding significant increases in soil fungal abundance following composting by earthworms^29^, which suggested that earthworms could stimulate soil fungal abundance^36,37^. Studies have also suggested that earthworm processing can lower soil (pathogenic) fungal population abundance, as earthworms can degrade the main component (chitin) of fungal cells^38^. Fungal hyphae become shortened and have a significantly lower abundance and diversity after being digested by earthworms^39^. However, in addition to the presence of earthworms, the experiment was conducted in an open system in which aquatic animals were present and the soil moisture content was stable. Earthworms and *M. albus* increase soil porosity, which drives the soil-atmosphere CO_2_ flux that is positively correlated with fungal abundance^40^. Studies have found that soil fungal abundance is positively correlated with relative moisture content when the latter is below 60%. Soil fungal abundance is negatively correlated with relative moisture content when the latter is above 60%^31^. In this study, the soil moisture content in the VEE-IPBP-treated plots was higher than that in the TPP-treated plots, although the soil moisture content in areas away from ditches was below 60%. This might explain why the soil moisture content in the VEE-IPBP-treated plots could facilitate fungal reproduction and increase fungal abundance. However, in open systems, interactions between multiple factors have directional effects on changes in soil fungal abundance and diversity. This makes predictions more difficult, as there is still uncertainty in examining changes in soil fungal abundance across time scales^41^.

The PCoA revealed that across time scales, the distributions of soil fungal community structure in the VEE-IPBP-treated and TPP-treated plots became more divergent. The results of the LEfSe analysis showed that there were 6 taxa in TPP16 and 13 in VEE16 that differed between the two planting systems. This could be due to the introduction of more species to the VEE-IPBP-treated plots, which altered the conditions in the TPP-treated plots and increased the number of influencing factors. Examples of crop types that can enrich fungal population structure and facilitate the coexistence of multiple fungal taxa include an intercropping system with maize, cowpea, and cotton that lasted for 2 consecutive years in a semiarid area in Brazil^42^; a 10-year continuous cropping and intercropping experiment with straw addition that involved planting a combination of flue-cured tobacco, wheat, and *B. rapa* in limestone yellow soil in northern Guizhou Province^32^; and a rice-duck integrated planting and rearing system^33^. In this study, environmental factors that influenced soil fungal taxa differed between planting systems and time scales. Soil available nitrogen, total phosphorus, available phosphorus, total nitrogen, and organic matter mainly affected *Pseudeurotium*, *Cosmospora*, *Cercophora*, and *Olpidium* in TPP10. Soil total potassium mainly affected *Petriella*, *Fusarium*, *Schizothecium*, and *Cylindrocarpon* in TPP13 and VEE13. Soil pH and moisture content mainly affected *Mortierella* and *Plectosphaerella* in TPP16 and VEE16. These findings are similar to those of Chen et al. (2015), in which 10 years after the implementation of different planting systems, the number of dominant *Ascomycota* spp. in the soil was reduced from 20 to 8^32^. This might be because the VEE-IPBP treatment altered the soil available nutrients, pH, moisture content, and aeration conditions^43^. For example, studies suggest that changes in nutrients such as available nitrogen, ammonia nitrogen, and nitrate nitrogen in black soil can alter fungal community structure and enhance fungal diversity and activity^44^, and soil pH is the most important factor affecting the soil fungal community composition^45^. Not only does the planting system alter the dominant taxa of soil fungi, it also affects the interaction networks between fungal taxa and their trophic modes. Similarly, mycorrhizal, endophytic, saprotrophic, and pathogenic fungi in forest soil could form compartmentalized (modular) networks of facilitative, antagonistic, and/or competitive interactions in belowground ecosystems^46^. In this study, it was also found that compared with TPP, the long-term VEE-IPBP treatment increased the proportions of the 3 independent trophic modes and 2 multitrophic modes (P–Sa and P–Sy) of soil fungi and reduced the proportions of S–S and fully mixed trophic modes. Fungal taxa, including *Cylindrocarpon*, *Plectosphaerella*, *Cosmospora*, and *Schizothecium*, which were dominant in the two planting systems, had different nodes in the interaction networks between planting systems. Our experiment was performed in an open system with many influencing factors. Thus, the experiment was unlike those in semi-enclosed or closed laboratories with strictly controlled conditions that more easily allow the reasons and influencing factors of particular phenomena to be explained. However, open experiments are representative of actual production, and the experimental findings obtained are of greater significance in guiding production practices. To better develop production practices, it is necessary to understand the contributions of internal and external factors as well as the effective nodes and pathways that can influence the system. Therefore, to make better use of agri-aquaculture planting systems, further research on the above issues in combination with controlled laboratory experiments is needed.

## Conclusions

In this study, high-throughput sequencing was used to study the community structure of soil fungi in a paddy-upland intercropping field. It was found that the combination mode increased the diversity indices of soil fungi and reduced the proportion of dominant species. On the temporal scale, the factors affecting soil fungi changed from nutritional factors to environmental factors, and the proportion of soil fungi with various nutritional functional types was altered.

## Materials and methods

### Overview of the experimental site

The experimental site was located on Chongming Island (31° 41′ 15″ N, 121° 54′ 00″ E) at an average elevation of 4 m above sea level. This island has a northern subtropical monsoon climate with a prevailing southeasterly wind. It has four distinct seasons with a hot, humid summer and a cold, humid winter and receives abundant light. Typhoons, heavy rains, and droughts are common climatic disasters. The average annual precipitation is 1003.7 mm, and precipitation is concentrated between April and September. The annual average air temperature is 15.3 °C, the accumulated temperature (≥ 10 °C) is 2559.60 degree-days, the frost-free period is 229 days, and the sunshine duration is 2104.0 h. The soil type is sand mixed with loess. The basic physical and chemical properties of the soil (0–20 cm) were as follows: soil organic matter 9.40 g/kg, total nitrogen 1.00 g/kg, total phosphorus 0.63 g/kg, total potassium 10.19 g/kg, available nitrogen 32.96 mg/kg, available phosphorus 10.33 mg/kg, available potassium 71.01 mg/kg, pH 8.73, and soil bulk density 1.37 g/cm^3^.

### Experimental design

The experiment began in June 2010. Two planting systems, a traditional planting platform (TPP) as the control and a VEE-IPBP system, were established^47^. For the VEE-IPBP system, both dry and wet fields were coupled within the same space and time (Fig. S1). In the dry field, dry-farmed vegetables (taro intercropped with broccoli, The collection of plant materials conforms to local and national standards and regulations, such as Code of practice for Production techniques of taro (DB42/T 1028-2014), Production technical practice for broccoli(GB/Z 26586-2011)) were planted, and earthworms (*Pheretima guillelmi*, native to Chongming Island) were reared. In the wet field, *Monopterus albus* (swamp eels) were reared. In this system, residue including the leaves and stalks of the vegetables were returned to and decomposed in the field as food for the earthworms. The earthworms were used as feed for *M. albus*. The faeces of the earthworms and *M. albus* provided nutrients for the vegetables. Hence, internal circulation of resources within the agricultural system was achieved^48^. There were 3 replicate plots for each treatment, and the plots were arranged in a randomized block. A commercial organic fertilizer consisting of organic matter (413.4 g/kg), nitrogen (N) (17.1 g/kg), phosphorous pentoxide (12.4 g/kg) and potassium oxide (12.3 g/kg) was applied as basal fertilizer at a dose of 18 t/hm^2^, and a compound fertilizer (N:P_2_O_5_:K_2_O = 15:15:15) (90% as base fertilizer and 10% as a topdressing material) was evenly sprayed at a dose of 375.0 kg/ha^2^ onto the vegetable field surface. The same commercial organic fertilizer was applied as basal fertilizer at a dose of 15 t/ha^2^, and compound fertilizer (N:P_2_O_5_:K_2_O = 15:15:15) (60% as base fertilizer and 40% as topdressing) was evenly sprayed at a dose of 750 kg/ha^2^ onto the cauliflower field surface. Earthworms (each weighing 3 g) were introduced at a density of 120 per m^2^ (the natural density was 60–80 per m^2^ in the surrounding vegetable field). The earthworm species used was William’s worm, the native species of Chongming Island in Shanghai. Over the course of the experiment, new earthworms were born, and old earthworms were killed or eaten by eels. In late winter and early spring when the temperature was above 6–10 °C, the number of earthworms in the field was investigated, and earthworms were removed or added so that the density of earthworms in the soil under the combination of planting and aquaculture was kept at approximately 120/m^2^. The results of the investigation in 2016 showed that the density of earthworms was 155/m^2^ in cultivated plots and 66/m^2^ in noncultivated plots. The yields and economic benefits from the two cropping patterns of earthworm, eel, taro and cauliflower are shown in Table S2. Compared with the yield, the economic benefit of the combined model was higher than that of the traditional model.

### Soil sample collection

Prior to the beginning of the experiment in June 2010, reference soil samples were collected from the vegetable fields. The samples were denoted as TPP10-1, TPP10-2, and TPP10-3. Soil samples were collected from the TPP- and VEE-IPBP-treated plots in November 2013 and November 2016. Soil samples from the TPP-treated plots were denoted as TPP13-1, TPP13-2, and TPP13-3 in 2013 and as TPP16-1, TPP16-2, and TPP16-3 in 2016. Soil samples from the VEE-IPBP-treated plots were denoted as VEE13-1, VEE13-2, and VEE13-3 in 2013 and as VEE16-1, VEE16-2, and VEE16-3 in 2016. Soil samples were collected from the surface layer (0–20 cm) in an “S”-shaped pattern using a 2-cm diameter stainless steel soil auger. In each replicate plot, samples were collected from 15 locations, mixed and stored in a sealed polyethylene bag, placed in a low-temperature container, and brought back to the laboratory. Impurities such as plant and animal remains were removed from the soil samples, after which they were mixed and sieved through a 0.84 mm-mesh sieve. Part of the sample was dried and analysed for the determination of basic soil physical and chemical properties, and the remainder was stored at − 80 °C for the determination of soil fungal community parameters.

### Test parameters and methods

#### Determination of soil physical and chemical properties

The soil organic matter (SOM) was determined by the potassium dichromate volumetric method. Total nitrogen (TN) was determined by the Kjeldahl method. Total phosphorus (TP) was determined by acid soluble aluminium antimony resistance colorimetry. Total potassium (TK) was determined by flame photometry using sodium hydroxide. Available nitrogen (AN) was determined by the Kjeldahl method. Available phosphorus (AP) was extracted by sodium bicarbonate and determined by aluminium antimony resistance colorimetry. Available potassium (AK) was determined by an acetic acid extraction flame photometer. The pH value was determined by the potentiometric method (the ratio of water to soil was 2.5:1). The moisture content was determined by the drying method^49^.

#### DNA extraction and PCR amplification

The total DNA was extracted from 1 g of the soil samples using the M5 Fungal Genomic DNA Kit (Mei5, Beijing, China), according to the manufacturer’s instructions. The DNA concentration was measured using NanoDrop 2000 (Thermo) and was found to be ≥ 20 ng μL^−1^. A region of the fungal ITS gene was amplified, using the primer pair ITS1F primer (5′-CTTGGTCATTTAGAGGAAGTAA-3′)^50^/ITS2 primer (5′-GCTGCGTTCTTCATCGATGC-3′)^51^. All samples were amplified in triplicates, and no-template controls were included at every step of the process. Triplicate PCR amplicons were pooled together, and detected on a 2% (w/v) agarose gel by electrophoresis. PCR products with a bright band were mixed in equal ratios and purified using AxyPrep DNA (Axygen). The purified PCR amplicon products were sequenced using the Illumina MiSeq PE300 platform (Illumina), Miseq Reagent kit v3 (600-cycle) (Illumina), by Shanghai Biozeron Biotech Co., Ltd (Shanghai, China).

### Data processing

#### Analysis of differences between treatments

The method that the authors compare the difference between two treatments with same planting age is One way ANOVA (SPSS, v 19, IBM).

#### OTU clustering analysis

Using the QIIME platform and referring to the fungal database NCBI (blastn v 2.3.0; evalue: 1e−50, http://www.ncbi.nlm.nih.gov/Traces/sra), operational taxonomic unit (OTU) representative sequences with 97% similarity were subjected to taxonomic analysis using the ribosomal database project (RDP) Bayesian classifier algorithm^52^.

#### Fungal diversity analysis

Species richness and diversity indices of fungal communities were analysed using Mothur software (version 1.30.1)^53^. Venn diagrams were generated, and principal coordinate analysis (PCoA) and community structure component analysis were performed using R^54^ (version 3.1.3, https://cran.r-project.org/ (vegan 2.4-4)).

#### Linear discriminant analysis effect size (LEfSe)

Communities or species contributing to significant differences in sample partitioning were determined using LEfSe software^55^ (https://github.com/biobakery/lefse; LEfSe 1.0).

#### Canonical correspondence analysis (CCA)

Relationships between environmental factors, samples, and fungal communities or their pairwise relationships were analysed and graphed using R^56^ (version 3.1.3, https://cran.r-project.org/ (vegan 2.4-4)).

#### Functional prediction using FUNGuild

Fungal ecological functions were analysed using FUNGuild software^57^ (https://github.com/UMNFuN/FUNGuild/; v1.1).

## Supplementary Information

Supplementary Figure S1.

Supplementary Table S1.

Supplementary Table S2.

## References

[1] Raina P, Kumar M, Singh M (2009). Mapping of soil degradation hazards by remote sensing in Hanumangarh district (western Rajasthan). J. Indian Soc. Remote Sens..

[2] Macdonald JM, Mcbride WD (2009). The Transformation of U.S. Livestock Agriculture: Scale, Efficiency, and Risks.

[3] Helgason BL, Walley FL, Germida JJ (2009). Fungal and bacterial abundance in long-term no-till and intensive-till soils of the northern Great Plains. Soil Sci. Soc. Am. J..

[4] An S, Couteau C, Luo F, Neveu J, Dubow MS (2013). Bacterial diversity of surface sand samples from the Gobi and Taklamaken deserts. Microb. Ecol..

[5] Hendrickson J, Sassenrath GF, Archer D, Hanson J, Halloran J (2008). Interactions in integrated US agricultural systems: The past, present and future. Renew. Agric. Food Syst..

[6] Hilimire K (2011). Integrated crop/livestock agriculture in the United States: A review. J. Sustain. Agric..

[7] Ghebremichael LT, Veith TL, Cerosaletti PE, Dewing DE, Rotz CA (2009). Exploring economically and environmentally viable northeastern US dairy farm strategies for coping with rising corn grain prices. J. Dairy Sci..

[8] Balkcom KS, Reeves DW, Kemble JM, Dawkins RA, Raper RL (2010). Tillage requirements of sweet corn, field pea, and watermelon following stocker cattle grazing. J. Sustain. Agric..

[9] Clark MS, Gage SH (1996). Effects of free-range chickens and geese on insect pests and weeds in an agroecosystem. Am. J. Altern. Agric..

[10] Sumberg J (2003). Toward a dis-aggregated view of crop–livestock integration in Western Africa. Land Use Policy.

[11] Halwart M, Gupta MV (2004). Culture of Fish in Rice Fields.

[12] Brito I, Goss MJ, Carvalho MD, Chatagnier O, Tuinen DV (2012). Impact of tillage system on arbuscular mycorrhiza fungal communities in the soil under Mediterranean conditions. Soil Tillage Res..

[13] Larkin RP (2003). Characterization of soil microbial communities under different potato cropping systems by microbial population dynamics, substrate utilization, and fatty acid profiles. Soil Biol. Biochem..

[14] Ling MA (2015). Effects of continuous potato cropping on the diversity of soil microorganisms. Chin. J. Eco-Agric..

[15] Wang Y (2010). Tillage, residue burning and crop rotation alter soil fungal community and water-stable aggregation in arable fields. Soil Tillage Res..

[16] Puget P, Angers DA, Chenu C (1998). Nature of carbohydrates associated with water-stable aggregates of two cultivated soils. Soil Biol. Biochem..

[17] Siokwu S, Anyanwn C (2012). Tolerance for heavy metals by filamentous fungi isolated from a sewage oxidation pond. Afr. J. Microbiol. Res..

[18] Nair A, Ngouajio M (2012). Soil microbial biomass, functional microbial diversity, and nematode community structure as affected by cover crops and compost in an organic vegetable production system. Appl. Soil Ecol..

[19] Hawksworth DL (2001). The magnitude of fungal diversity: The 1.5 million species estimate revisited. Mycol. Res..

[20] Manka, M. & Fruzynskajozwiak, D. Biocontrol of greenhouse carnation Fusarium wilt with saprophytic forest soil fungi[J]. (1996).

[21] Hjort K, Presti I, Elväng A, Marinelli F, Sjöling S (2014). Bacterial chitinase with phytopathogen control capacity from suppressive soil revealed by functional metagenomics. Appl. Microbiol. Biotechnol..

[22] Liu Y (2004). A Broad-Spectrum Organophosphorus Hydrolase from Fungus.

[23] Alguacil MM (2011). The application of an organic amendment modifies the arbuscular mycorrhizal fungal communities colonizing native seedlings grown in a heavy-metal-polluted soil. Soil Biol. Biochem..

[24] Russelle MP, Entz MH, Franzluebbers AJ (2007). Reconsidering integrated crop–livestock systems in North America. Agron. J..

[25] Funes-Monzote F (2008). Farming Like We are Here to Stay: The Mixed Farming Alternative for Cuba.

[26] Brunson MW, Huntsinger L (2008). Ranching as a conservation strategy: Can old ranchers save the new west?. Rangel. Ecol. Manage..

[27] Mandić L, Đukić D, Stevović V (2004). The number of soil fungi and maize productivity in different fertilizing conditions. Acta Agric. Serbica.

[28] Zhu C (2016). Impacts of fertilization regimes on arbuscular mycorrhizal fungal (AMF) community composition were correlated with organic matter composition in maize rhizosphere soil. Front. Microbiol..

[29] Wang Y (2015). Community dynamics of arbuscular mycorrhizal fungi in high-input and intensively irrigated rice cultivation systems. Appl. Environ. Microbiol..

[30] Cruz-Paredes C, Wallander H, Kjøller R, Rousk J (2017). Using community trait-distributions to assign microbial responses to pH changes and Cd in forest soils treated with wood ash. Soil Biol. Biochem..

[31] Clegg CD (2006). Impact of cattle grazing and inorganic fertiliser additions to managed grasslands on the microbial community composition of soils. Appl. Soil. Ecol..

[32] Chen D (2015). Influence of cropping system on enzyme activities and fungal communities in soil. Acta Pratacult. Sin..

[33] Liao Y (2019). Analysis of population diversity of fungi and bacteria in rice rhizosphere soil under rice-duck farming model. J. South. Agric..

[34] Averill C, Turner BL, Finzi AC (2014). Mycorrhiza-mediated competition between plants and decomposers drives soil carbon storage. Nature.

[35] Worrich A (2017). Mycelium-mediated transfer of water and nutrients stimulates bacterial activity in dry and oligotrophic environments. Nat. Commun..

[36] Groffman PM, Bohlen PJ, Fisk MC, Fahey TJ (2004). Exotic earthworm invasion and microbial biomass in temperate forest soils. Ecosystems.

[37] Feng H, Xia W, Huixin L (2005). Effects of earthworms on soil microbial biomass carbon in rice-wheat rotation agro-ecosystem. Acta Pedol. Sin..

[38] Shan, J. *et al.**Enhanced Safety of Vehicles Conference* 9–17.

[39] Byzov BA, Khomyakov NV, Kharin SA, Kurakov AV (2007). Fate of soil bacteria and fungi in the gut of earthworms. Eur. J. Soil Biol..

[40] Sauze J (2017). The interaction of soil phototrophs and fungi with pH and their impact on soil CO2, CO18O and OCS exchange. Soil Biol. Biochem..

[41] Rillig MC (2019). The role of multiple global change factors in driving soil functions and microbial biodiversity. Science.

[42] Garrido MDS (2012). Occurrence of arbuscular mycorrhizal fungi after organic fertilization in maize, cowpea and cotton intercropping systems. Acta Sci. Agron..

[43] Lanzén A (2015). The community structures of prokaryotes and fungi in mountain pasture soils are highly correlated and primarily influenced by pH. Front. Microbiol..

[44] Ding JL (2017). Structure of soil fungal communities under long-term inorganic and organic fertilization in black soil of Northeast China. J. Plant Nutr. Fertil..

[45] Zhang T, Wang N-F, Liu H-Y, Zhang Y-Q, Yu L-Y (2016). Soil pH is a key determinant of soil fungal community composition in the Ny-Ålesund Region, Svalbard (High Arctic). Front. Microbiol..

[46] Toju H, Kishida O, Katayama N, Takagi K (2016). Networks depicting the fine-scale co-occurrences of fungi in soil horizons. PLoS ONE.

[47] Zheng X, Li S, Yuan D, He Q, Zhang J (2012). Effects of biological tillage on soil nutrients and enzyme activities in vegetable fields. Plant Nutr. Fertil. Sci..

[48] Zheng X (2018). Effects of a vegetable-eel-earthworm integrated planting and breeding system on bacterial community structure in vegetable fields. Sci. Rep..

[49] Lu R (1999). Analytical Methods of Soil Agricultural Chemistry.

[50] Gardes M, Bruns TD (1993). ITS primers with enhanced specificity for basidiomycetes—Application to the identification of mycorrhizae and rusts. Mol. Ecol..

[51] White TJ, White TJ (1990). Amplification and direct sequencing of fungal ribosomal RNA genes for phylogenetics. Pcr Protocols.

[52] Wang Q, Garrity GM, Tiedje JM, Cole JR (2007). Naive Bayesian classifier for rapid assignment of rRNA sequences into the new bacterial taxonomy. Appl. Environ. Microbiol..

[53] Schloss PD, Gevers D, Westcott SL (2011). Reducing the effects of PCR amplification and sequencing artifacts on 16S rRNA-based studies. PLoS ONE.

[54] Fouts DE (2012). Next generation sequencing to define prokaryotic and fungal diversity in the bovine rumen. PLoS ONE.

[55] Zhang C (2013). Structural modulation of gut microbiota in life-long calorie-restricted mice. Nat. Commun..

[56] Sheik CS (2012). Exposure of soil microbial communities to chromium and arsenic alters their diversity and structure. PLoS ONE.

[57] Nguyen NH (2016). FUNGuild: An open annotation tool for parsing fungal community datasets by ecological guild. Fungal Ecol..

