# Supplementary material for: Effects of long-term integrated agri-aquaculture on the soil fungal community structure and function in vegetable fields
Source: Sci Rep. 2021 May 24;11:10813. doi: 10.1038/s41598-021-90109-6 (PMC8144417; doi:10.1038/s41598-021-90109-6)
Supplement: Supplementary file 1 — Supplementary Figure S1. [file 41598_2021_90109_MOESM1_ESM.docx]

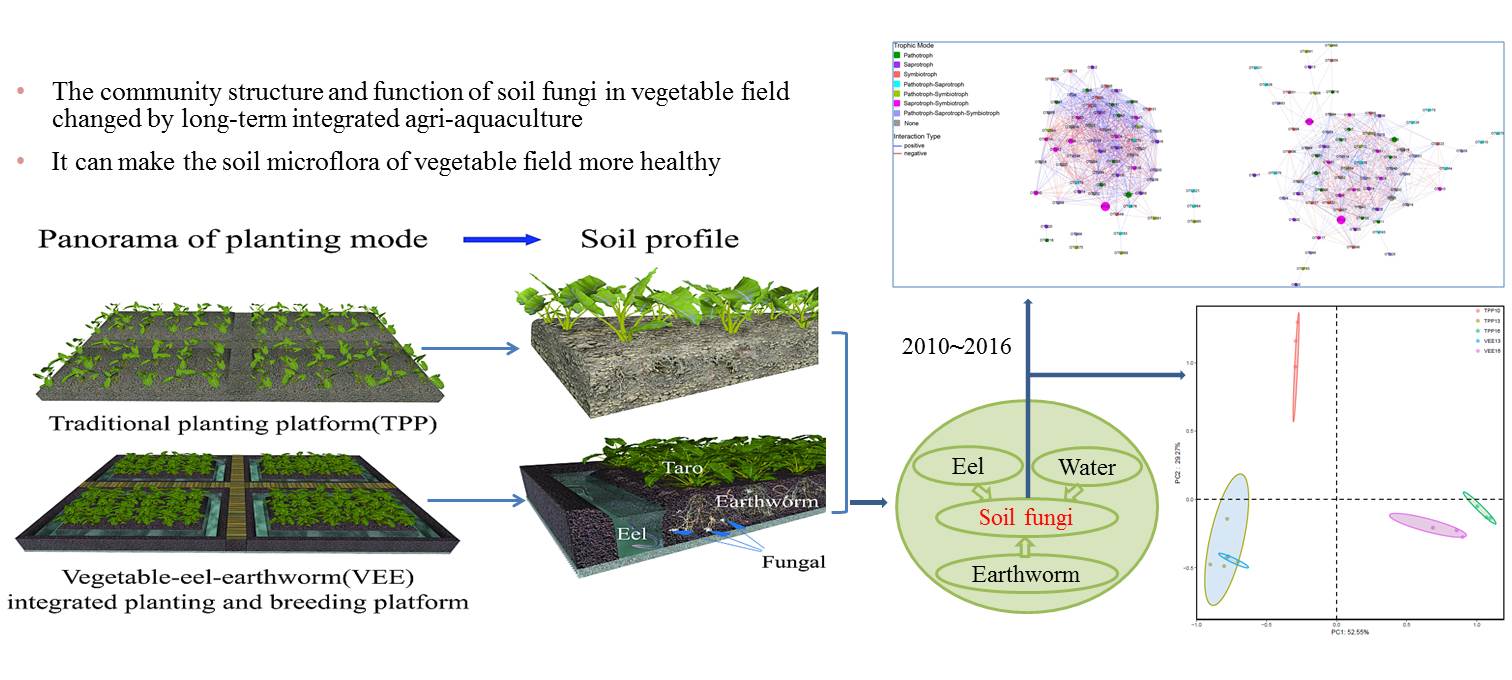


**Fig S1:** The difference between the platform of integrated planting and breeding and the traditional, and the influence of the platform of integrated planting and breeding on soil fungi.
