# Supplementary material for: Effects of long-term integrated agri-aquaculture on the soil fungal community structure and function in vegetable fields
Source: Sci Rep. 2021 May 24;11:10813. doi: 10.1038/s41598-021-90109-6 (PMC8144417; doi:10.1038/s41598-021-90109-6)
Supplement: Supplementary file 2 — Supplementary Table S1. [file 41598_2021_90109_MOESM2_ESM.docx]

**Table 1. Statistics of trimmed sequences and length distribution of valid sequences (a).**

| **Trimmed** | **Samples** | **Sequences** | **Bases (bp)** | **Average Length (bp)** |
| --- | --- | --- | --- | --- |
| ITS1F_ITS2R | 15 | 561254 | 133924507 | 238.62 |

**Table 1. Statistics of trimmed sequences and length distribution of valid sequences (b).**

| **Length (bp)** | **Sequences** | **Percent (%)** |
| --- | --- | --- |
| 1-200 | 325 | 0.06 |
| 201-250 | 417080 | 74.31 |
| 251-300 | 131974 | 23.51 |
| 301-350 | 11875 | 2.12 |
