# Supplementary material for: Effects of long-term integrated agri-aquaculture on the soil fungal community structure and function in vegetable fields
Source: Sci Rep. 2021 May 24;11:10813. doi: 10.1038/s41598-021-90109-6 (PMC8144417; doi:10.1038/s41598-021-90109-6)
Supplement: Supplementary file 3 — Supplementary Table S2. [file 41598_2021_90109_MOESM3_ESM.docx]

**Table 2 Comparison of yield and benefit of unit farmland under two planting modes**

| Treatment | Production（kg/667m^2^） | | | | | Economic benefits（rmb/667m^2^） | | | |
| --- | --- | --- | --- | --- | --- | --- | --- | --- | --- |
|  | Broccoli | Taro | Eel | Earthworm | Broccoli | Taro | Eel | Earthworm | Total |
| TPP10 | 2356.2 | 906.2 | / | / | 2356.2 | 5437.2 | / | / | 5437.2 |
| TPP13 | 2597.3 | 955.7 | / | 75.4 | 2597.3 | 5734.2 | / | 1508.0 | 7242.2 |
| VEE13 | 2880.8 | 960.4 | 9.6 | 155.5 | 2880.8 | 5762.4 | 1152.0 | 3110.0 | 10024.4 |
| TPP16 | 2708.1 | 902.8 | / | 60.8 | 2708.1 | 5416.8 | / | 1216.0 | 6632.8 |
| VEE16 | 3003.6 | 1012.6 | 13.7 | 183.7 | 3003.6 | 6075.6 | 1644.0 | 3674.0 | 11393.6 |

Note：From 2010 to 2016, the average market price of agricultural products, The average value of cauliflower is 1 yuan / kg, taro is 6 yuan / kg, earthworm is 20 yuan / kg, eel is 120 yuan / kg.
